# Overestimation of Crop Root Biomass in Field Experiments Due to Extraneous Organic Matter

**DOI:** 10.3389/fpls.2017.00284

**Published:** 2017-03-01

**Authors:** Juliane Hirte, Jens Leifeld, Samuel Abiven, Hans-Rudolf Oberholzer, Andreas Hammelehle, Jochen Mayer

**Affiliations:** ^1^Department of Natural Resources and Agriculture, Institute for Sustainability Sciences, AgroscopeZurich, Switzerland; ^2^Department of Geography, University of ZurichZurich, Switzerland; ^3^Landesbetrieb Landwirtschaft HessenFriedberg, Germany

**Keywords:** dead roots, debris, residues, remnants, maize, arable farming, agricultural management, organic inputs

## Abstract

Root biomass is one of the most relevant root parameters for studies of plant response to environmental change, soil carbon modeling or estimations of soil carbon sequestration. A major source of error in root biomass quantification of agricultural crops in the field is the presence of extraneous organic matter in soil: dead roots from previous crops, weed roots, incorporated above ground plant residues and organic soil amendments, or remnants of soil fauna. Using the isotopic difference between recent maize root biomass and predominantly C3-derived extraneous organic matter, we determined the proportions of maize root biomass carbon of total carbon in root samples from the Swiss long-term field trial “DOK.” We additionally evaluated the effects of agricultural management (bio-organic and conventional), sampling depth (0–0.25, 0.25–0.5, 0.5–0.75 m) and position (within and between maize rows), and root size class (coarse and fine roots) as defined by sieve mesh size (2 and 0.5 mm) on those proportions, and quantified the success rate of manual exclusion of extraneous organic matter from root samples. Only 60% of the root mass that we retrieved from field soil cores was actual maize root biomass from the current season. While the proportions of maize root biomass carbon were not affected by agricultural management, they increased consistently with soil depth, were higher within than between maize rows, and were higher in coarse (>2 mm) than in fine (≤2 and >0.5) root samples. The success rate of manual exclusion of extraneous organic matter from root samples was related to agricultural management and, at best, about 60%. We assume that the composition of extraneous organic matter is strongly influenced by agricultural management and soil depth and governs the effect size of the investigated factors. Extraneous organic matter may result in severe overestimation of recovered root biomass and has, therefore, large implications for soil carbon modeling and estimations of the climate change mitigation potential of soils.

## Introduction

Plant roots play a crucial role in carbon (C) and nutrient cycling, they promote the formation and structural stability of soils, and shape entire communities of soil organisms. Roots are therefore key players in many ecosystem processes (Philippot et al., 2013; Bardgett et al., 2014). At the same time, roots are highly responsive to their environment by unfolding their physical and functional characteristics with respect to plant growth conditions (Gregory, 2006; Hodge et al., 2009). The most commonly investigated root parameter in studies of plant response to environmental change is root biomass as it is closely linked to the energy investment of plants in their root systems or, in other words, the amount of C that is allocated below ground (Fageria, 2013). This makes it one of the most relevant root parameters for soil C modeling and for identifying efficient climate change mitigation options (Bolinder et al., 2007; Paustian et al., 2016).

In agricultural systems with annual crops, root biomass is closely resembled by the entirety of crop roots at the time of sampling since root mortality during the plant’s life cycle is comparably low (Pritchard and Rogers, 2000). Roots are often categorized in ‘coarse’ and ‘fine’ roots based on diameters of more than or maximum 2 mm, respectively, when the functional duality of the root system with both longer- and shorter-lived roots is of relevance (Smithwick et al., 2014) as in studies of root decomposition and C sequestration in soil (Silver and Miya, 2001; Zhang and Wang, 2015).

The most common method to quantify root biomass in agricultural fields comprises destructive volumetric soil sampling, separation of fresh roots from soil by either manual or automated wet-sieving, and extrapolation of the dried root weight to the study area (Gregory, 2006). Methodological specifications regarding sampling device, depth or position and washing agent, duration of washing or sieve mesh size vary widely between studies, resulting in large differences of recovered root biomass (Oliveira et al., 2000).

Another source of error in root studies is the presence of extraneous organic matter (Ottman and Timm, 1984), hereafter abbreviated with EOM. Although frequently addressed in the literature, various expressions have been used to describe this form of organic matter that is recovered together with recent crop roots: “dead” or “decaying roots” (Schuurman and Goedewaagen, 1971; Gregory et al., 1978; Ward et al., 1978; Moran et al., 2000; Pierret et al., 2005), “other organic matter” (Gregory et al., 1978; Ward et al., 1978), “debris” (Dowdy et al., 1998; Livesley et al., 1999; Benjamin and Nielsen, 2004), “non-root residues” (Pietola and Smucker, 2006) and “remnants” (Watt et al., 2008). The diversity of terms may be the result of various components that can accrue as EOM in soil: dead roots from previous crops, weed roots, incorporated above ground plant residues and organic soil amendments (mulch, manure, slurry), or remnants of soil fauna.

There are mainly two factors that influence the amount of EOM in root samples: (i) Conditions in the field such as soil characteristics or agricultural management drive EOM accrual in soil (Watt et al., 2008) and (ii) methodological specifications of sampling in the field and sample processing in the lab determine how much of this EOM present in soil is finally collected and retained together with the crop roots (Livesley et al., 1999; Pietola and Smucker, 2006).

(i) Agricultural management affects EOM accrual in soil in a complex way and many overlying factors contribute to the overall effect. Organic management is characterized by regular application of animal manure and frequent integration of green manures and cover crops in the crop rotation whereas conventional management can also imply stockless farming without residue retention (Lorenz and Lal, 2016). However, organic yields are typically lower by 20–25% than conventional yields (Seufert et al., 2012; Niggli et al., 2016) while below ground crop biomass does not seem to differ significantly between farming systems (Chirinda et al., 2012; Taghizadeh-Toosi et al., 2016). Additionally, above and below ground plant material can also originate from weed which is usually more abundant in organic than in conventional systems (Hawes et al., 2010; Rotchés-Ribalta et al., 2016). Management also determines the quality and fate of these inputs in soil (Kong et al., 2005; Leifeld and Fuhrer, 2010; Gattinger et al., 2012) as higher stability of organic soil amendments (Romanyà et al., 2012) and less readily available mineral nitrogen (Khan et al., 2007; Cai et al., 2015) may entail lower decomposition rates in organic than in conventional soils. However, the direction and size of the net effect of these management-related factors on EOM accrual in soil is unclear.

(ii) Methodological specifications affect the proportion of EOM finally retained in samples in several ways. In the field, recent crop root biomasses and organic inputs that contribute to EOM are often differently distributed in soil. Root biomasses of row crops, on the one hand, concentrate in the topsoil within rows (Fan et al., 2016; Frasier et al., 2016) and are inherently non-uniformly and non-randomly distributed (Allmaras and Nelson, 1971). Organic soil amendments, on the other hand, are usually homogeneously applied to the soil surface and occur mainly within the lower half of the plow layer after incorporation into soil (Schneider et al., 2006; Norén, 2009). The effect of EOM on total sample mass is therefore strongly related to sampling depth and position within or between crop rows. In the lab, the choice of sieve mesh size for root washing influences EOM retention and the practice of sample cleansing from EOM particles determines the amount of EOM that finally remains in the root sample. Retention of EOM with coarse sieves (≥1 mm mesh) is small but increases considerably with decreasing mesh size (≤0.5 mm mesh; Livesley et al., 1999; Koteen and Baldocchi, 2013). Samples are therefore usually cleansed from EOM particles either by classical visual distinction, which uses differences in shape, elasticity, and color between recent root biomass and EOM (Schuurman and Goedewaagen, 1971; Watt et al., 2008), or by automated image analysis of sample scans (Benjamin and Nielsen, 2004; Pietola and Smucker, 2006), often combined with vital staining of the roots (Richner et al., 2000). However, neither of those methods result in complete exclusion of EOM from root samples (Pietola and Smucker, 2006; Watt et al., 2008).

To our knowledge, the proportion of actual recent crop root biomass in root samples from agricultural fields has never been quantified. One of the most challenging aspects is the distinction between recent crop roots and EOM. The natural C isotope composition can serve as a distinction criterion when the respective δ^13^C values are known and sufficiently different from each other as in the case of C3- and C4-plant derived material (O’Leary, 1988).

The objectives of our study were, therefore, (i) to determine the proportion of actual recent crop root biomass C of total C in root samples from an agricultural field using the isotopic difference between crop root biomass and EOM, (ii) to evaluate the effects of agricultural management, sampling depth and position, and sieve mesh size on those proportions, and (iii) to quantify the success rate of manual exclusion of EOM from root samples.

## Materials and Methods

### Experimental Site and Management Treatments

Root samples were taken in 2013 on silage maize (*Zea mays* L. var. Colisee) plots of the Swiss long-term field trial DOK [47°50′25″ N, 7°53′93″ E; 308 m above sea level; mean annual temperature 10.5°C; mean annual precipitation 842 mm for 1981–2010; more details in Leifeld et al. (2009) and Mayer et al. (2015)]. In brief, the DOK trial compares organic and conventional farming with two fertilization levels, respectively, according to Swiss standards and has a distinct crop rotation of maize followed by 6 years of exclusively C3-plants (Supplementary Table S1). The soil is a haplic Luvisol with, on average, 12% sand, 72% silt, and 16% clay. We chose two bio-organic treatments with half and full fertilization (BIOORG1 and BIOORG2) and a mixed conventional treatment with full fertilization (CONFYM2), which followed an intensity gradient with respect to nutrient inputs (**Table 1**). Weed control was done chemically in CONFYM2 and manually in BIOORG1 and BIOORG2. However, weed could grow to some extent in the bio-organic treatments and the community was composed to the largest part of C3-plants [<4% of total weed abundance could be attributed to *Amaranthus blitoides* S. Watson and *Echinochloa crus-galli* (L.) Beauv in 2009; R. Rotchés-Ribalta, personal communication]. The treatments were replicated four times in the field, providing a total of 12 experimental plots.

**Table 1:** Fertilizer types, applied fertilizer nutrients, stand densities at harvest, and yields of silage maize of organically (BIOORG) and conventionally (CONFYM) managed plots of the DOK trial in 2013 (values in brackets: average of 2006–2012).

Treatment	Fertilizer		Applied nutrients [kg ha^-1^]				Stand density [plants m^-2^]	Yield [t ha^-1^]^b^
			
	Type^a^	Mass [t ha^-1^]^b^	*N*_total_	*N*_min_	*P*	*K*		
BIOORG1	FYM + SL	2.7 + 0.4	91 (43)	18 (11)	23 (12)	183 (108)	8.5	12.5
BIOORG2	FYM + SL	5.3 + 0.8	182 (86)	36 (22)	47 (25)	365 (216)	8.8	13.2
CONFYM2	FYM + SL	6.4 + 0.6	225 (199)	92 (129)	40 (38)	299 (251)	8.9	18.6
	+ mineral		110 (62)	110 (62)	0 (15)	0 (66)


*^a^ FYM: farmyard manure; SL: slurry. ^b^ dry matter.*

Before maize was sown in May 2013, the preceding two-and-a-half-years old grass-clover ley was harvested and plowed. The row width of maize was 0.75 m and the approximate plant distance within rows was 0.15 m. The precise sowing densities were 10.5 plants m^-2^ for the organic and 9.5 plants m^-2^ for the conventional treatments to compensate for different sprouting success of organic and conventional maize. This resulted in similar stand densities for all treatments at harvest (**Table 1**). The maize was harvested at the end of September 2013. Fertilization details and harvest parameters for maize 2013 are given in **Table 1**.

### Sampling and Sample Processing

**FIGURE 1:**
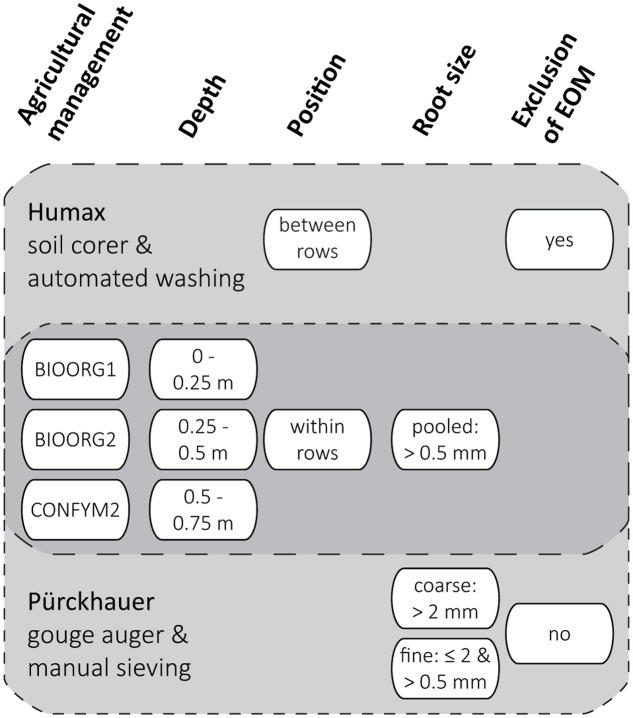
**Sampling and sample processing scheme for the determination of proportions of maize root biomass C in root samples by two methods (Humax and Pürckhauer).** Levels (white ovals) of analyzed factors (bold headings) were analyzed for both Humax and Pürckhauer samples (dark gray area) or for either Humax or Pürckhauer samples only (light gray areas).

After harvest, we took soil cores from each experimental plot from three soil depths (0–0.25, 0.25–0.5, 0.5–0.75 m) with two different methods, respectively. We used a Humax core sampler (ϕ 50 mm; Martin Burch AG, Switzerland) to take one core within and one half way between maize rows and a Pürckhauer gouge auger (ϕ 30 mm; Eijkelkamp, Netherlands) to take four cores within rows only. Multiple soil cores from the same experimental plot and depth and taken with the same method were pooled and all samples were stored at 4°C for a maximum of 3 weeks before processing. The sampling and sample processing scheme is presented in **Figure 1**.

Roots of the Humax samples were obtained by automated wet sieving using a root washer (Hydropneumatic Elutriation System GVF 13000; Gillison’s Variety Fabrication Inc., USA): The field-fresh soil cores were dispersed by a high energy hydrovortex at a water pressure of approx. 3.5 kg cm^-2^ for 10 min before organic material was separated from the mineral fraction by flotation and recovered on a 0.5 mm mesh (Smucker et al., 1982). Roots of the Pürckhauer samples were obtained by manual sieving using hand sieves in a two-step procedure: The field-fresh soil samples were sieved through a 2 mm mesh, the recovered roots were rinsed under running tap water and the sieved soil was homogenized and air-dried for 48 h. Subsamples of 240 g dried soil were then dispersed in 300 mL tap water by overhead-shaking in 1 L PE-bottles for 20 min and subsequently wet sieved through a 0.5 mm mesh. The sieve residue was rinsed under running tap water at a water pressure of approximately 1 kg cm^-2^ and remaining soil aggregates were carefully broken with a soft rubber spatula. When visibly free of soil, the residue was then transferred into a plastic bowl and organic material was separated from the mineral fraction by repeated decantation.

We defined the resulting root size classes as pooled (>0.5 mm) roots (Humax samples) and coarse (>2 mm) and fine (≤2 and >0.5 mm) roots (Pürckhauer samples). An attempt to exclude EOM from the samples by visual distinction was only made for the pooled roots: The washed roots were spread in an aluminum dish and EOM was identified based on shape and structure (pieces of above ground plant residues and manure often have a rectangular shape and a coarser surface structure than roots) and color and elasticity (old roots are darker and less elastic than recent roots) and removed from the samples using tweezers (Schuurman and Goedewaagen, 1971). All roots were dried at 60°C and ground with a mixer mill (MM200; Retsch, Germany) for total C and ^13^C analysis. We used fine roots of the 2012 grass-clover ley from the topsoil (0–0.25 m) of the same plots to derive the δ^13^C value of EOM in 2013 (see below). Those roots were obtained, processed, and analyzed in October 2012 in the same way as the Pürckhauer maize roots in 2013.

### Total C and ^13^C Analysis

Total C and the ^13^C/^12^C ratios of the roots were determined by isotope ratio mass spectrometry (IRMS) using an elemental analyzer (EA 1110; Carlo Erba, Italy) coupled with a mass spectrometer (Delta S; Thermo Finnigan, Germany). The δ^13^C value was expressed relative to the international V-PDB standard and the analytical precision, which is the standard deviation (SD) of the measured results of the working standard (plant biomass), was 0.2‰.

### Calculation of the Proportion of Maize Root Biomass C

We determined the proportions of maize root biomass C in the root samples by calculating a two-pool mixing model based on mass balance (Fry, 2008):

$$f_{RBC}=\frac{\left(\delta^{13}C_{s}-\delta^{13}C_{EOM}\right)}{\left(\delta^{13}C_{RB}-\delta^{13}C_{EOM}\right)}$$

where *f*_RBC_ is the mass fraction of maize root biomass C of total C in the sample and δ^13^C_s_, δ^13^C_RB_, and δ^13^C_EOM_ are the δ^13^C values of the sample, maize root biomass, and EOM, respectively. Accordingly, *f*_RBC_ and the mass fraction of EOM C of total C in the sample sum up to 1.

**FIGURE 2:**
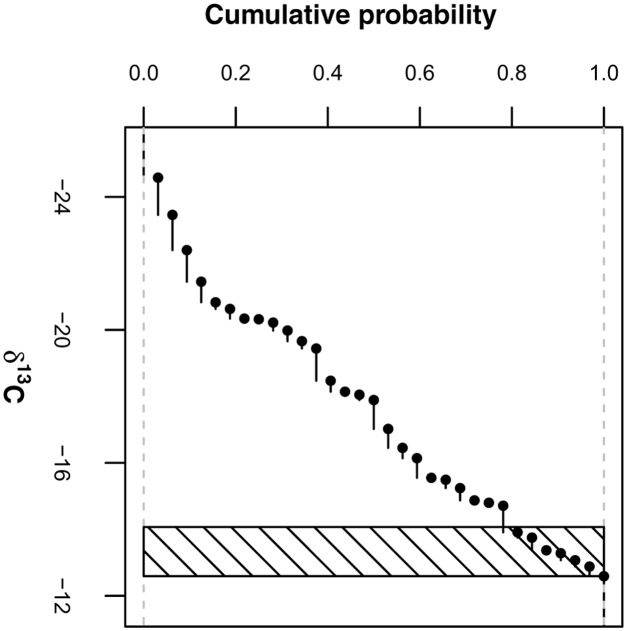
**Empirical cumulative distribution of δ^13^C values of root samples (>2 mm) containing maize coarse roots and non-maize EOM.** Values within shaded area (i.e., values larger than 0.8 quantile) are treated as δ^13^C values of pure maize root biomass.

We estimated the δ^13^C source values in this two-pool mixing model, δ^13^C_RB_ and δ^13^C_EOM_, as follows: δ^13^C_RB_ was derived from the maize coarse roots of 2013 by averaging the δ^13^C values of presumably pure maize roots (represented by values larger than the 0.8 quantile of all coarse roots; **Figure 2**) irrespective of agricultural management treatment and soil depth [δ^13^C_RB_ = -13.3 ± (SD) 0.5‰; *n* = 7; differences between treatments and depths not significant]. δ^13^C_EOM_ was derived from the 2012 grass-clover ley dataset by averaging the δ^13^C values of the fine root samples irrespective of treatment [δ^13^C_EOM_ = -29.3 ± (SD) 0.3‰; *n* = 12; difference between treatments not significant]. Due to methodological reasons, we expected that these samples were similarly affected by EOM in 2012, strongly suggesting that their composition resembled the composition of EOM in the maize root samples of 2013 for the most part. Proportion values for the pooled root size class of the Pürckhauer samples were generated by calculating weighted averages of the proportion values of coarse and fine root samples with respect to their mass ratios determined in a parallel study (Supplementary Data).

### Statistics

The data were fitted to linear mixed models to account for the multi-level order of explanatory variables and non-orthogonality of data (five out of 144 observations missing). Differences of the means were determined for the proportions of maize root biomass C by analysis of variance (ANOVA) with Kenward-Roger approximation for degrees of freedom and were considered as significant at *p* < 0.05. Due to the incomplete factorial design of the sampling and sample processing we used the complete data set only to test for differences between agricultural management treatments and soil depths. In addition, we used three different data subsets to test for differences between (1) sampling positions (row, inter-row), (2) root size classes (coarse, fine), and (3) EOM exclusion practices (no, yes). Subset (1) comprised all Humax samples, subset (2) comprised all Pürckhauer samples, while subset (3) was a combination of Humax and Pürckhauer samples that matched the criteria of same position (row) and same root size class (pooled) only. As our selection of management systems and fertilization levels was not fully crossed (BIOORG: levels 1 and 2; CONFYM: level 2 only) we analyzed the three treatments as independent levels and, thereby, ignored the strip-/split-character of the field design. We used the software R version 3.3.0 (R Core Team, 2016) and the R packages “lme4” (Bates et al., 2016), “lmerTest” (Kuznetsova et al., 2016), and “pbkrtest” (Halekoh and Højsgaard, 2016) for statistical analyses and the R package “lattice” (Sarkar, 2015) for data visualization.

## Results

Our 139 root samples from the field had 60% maize root biomass C or, in other words, 40% EOM C averaged over management treatments, sampling depths and positions, root size classes, and EOM exclusion practices. The proportions of maize root biomass C were highly variable between individual samples and ranged from 5 to 100%.

When taking agricultural management into consideration, the proportions of maize root biomass C averaged 55, 59, and 66% for BIOORG1, BIOORG2, and CONFYM2, respectively, indicating a slight increase with increasing management intensity. This difference was statistically not significant (*p* = 0.342). However, the overall trend recurred in all three soil depths individually (**Table 2**).

**Table 2:** Mean values and standard errors of proportions of maize root biomass C in root samples from different soil depths taken on organically (BIOORG) and conventionally (CONFYM) managed plots of the DOK trial.

Depth [m]		BIOORG1 n.s.	BIOORG2 n.s.	CONFYM2 n.s.
0–0.25 a		0.44 ± 0.07 (16)	0.48 ± 0.06 (16)	0.64 ± 0.07 (16)
0.25–0.5 ab		0.56 ± 0.06 (15)	0.61 ± 0.07 (16)	0.64 ± 0.06 (15)
0.5–0.75 b		0.64 ± 0.06 (16)	0.67 ± 0.08 (14)	0.71 ± 0.06 (15)


*Differing letters: significant differences between least squares means of proportion values across soil depths at *p* < 0.05. n.s.: differences between least squares means of proportion values across management systems not significant. Numbers in brackets: sample sizes.*

**FIGURE 3:**
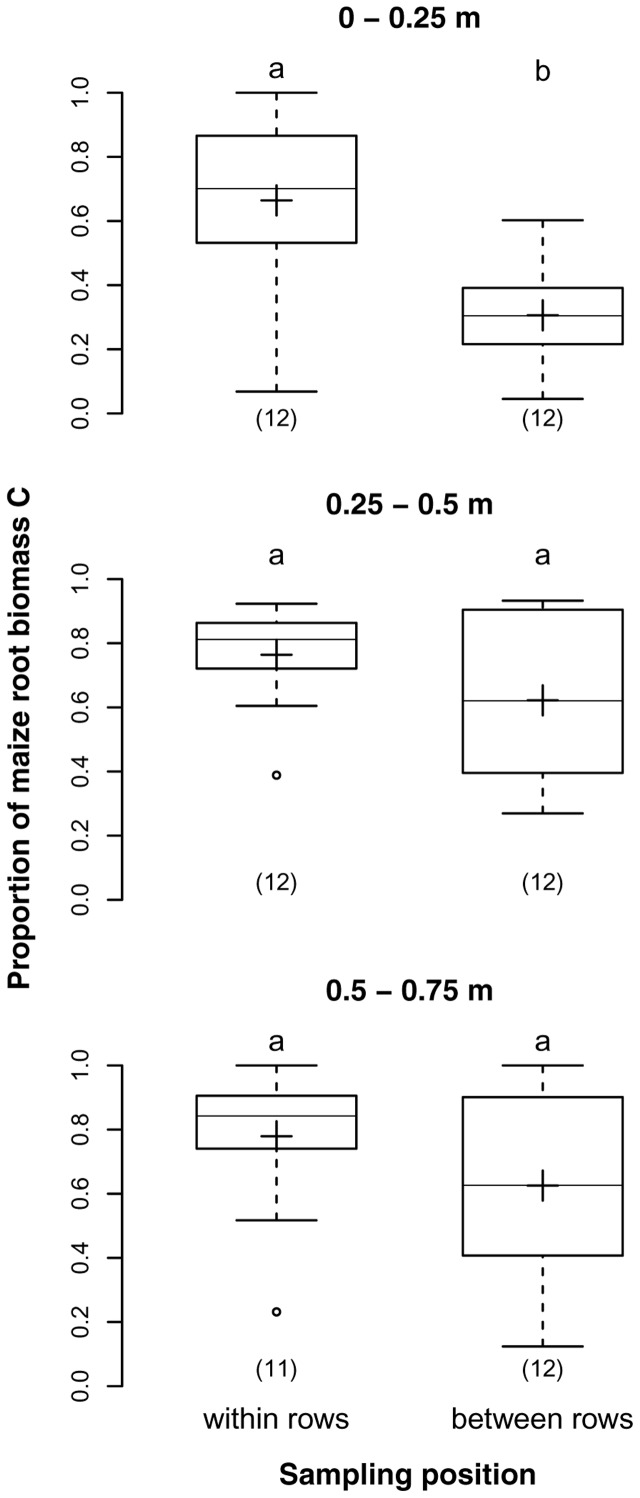
**Proportions of maize root biomass C in root samples from different soil depths within and between maize rows of the DOK trial averaged over management treatment [Humax samples: pooled root size class (>0.5 mm), after EOM exclusion].** Solid line, plus sign, boxes, and whiskers: median, mean, interquartile range (IQR), and 1.5 × IQR, respectively. Differing letters: significant differences between least squares means of proportion values across soil depths and sampling positions at *p* < 0.05. Numbers in brackets: sample sizes.

Both sampling depth and position had a highly significant effect on the proportions of maize root biomass C in our samples. The average proportions for the three sampling depths 0–0.25, 0.25–0.5, and 0.5–0.75 m were 52, 60, and 68%, respectively, reflecting a significant increase with depth (*p* = 0.007). Samples from the two sampling positions within and between rows had on average 73 and 52% maize root biomass C, respectively (*p* < 0.001). The difference was particularly prominent in 0–0.25 m depth, were the proportion of maize root biomass C was twice as high in samples taken in the row than between rows (**Figure 3**), but insignificant below 0.25 m depth as revealed by the significant interaction of sampling depth and position (*p* = 0.028).

**FIGURE 4:**
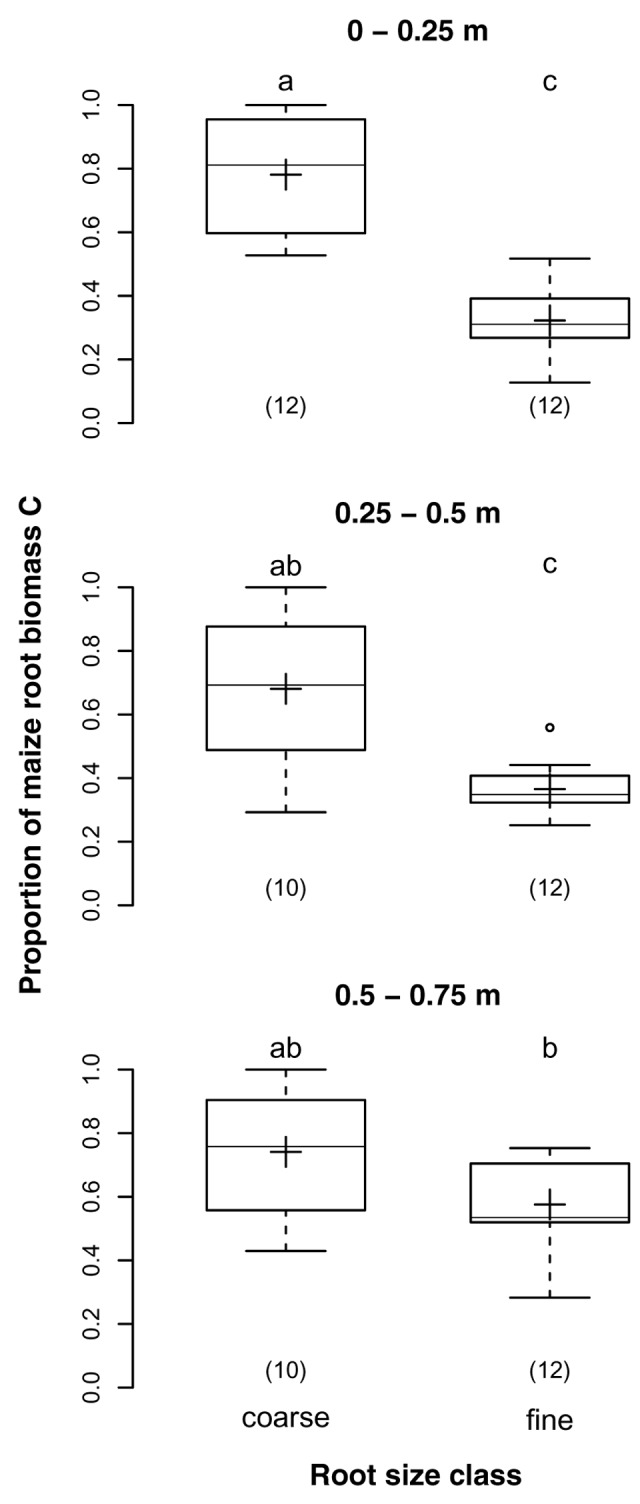
**Proportions of maize root biomass C in coarse (>2 mm) and fine (>0.5 and ≤2 mm) root samples from different soil depths of the DOK trial averaged over management treatment (Pürckhauer samples: within maize rows, without EOM exclusion).** Solid line, plus sign, boxes, and whiskers: median, mean, IQR, and 1.5 × IQR, respectively. Differing letters: significant differences between least squares means of proportion values across soil depths and root size classes at *p* < 0.05. Numbers in brackets: sample sizes.

The proportions of maize root biomass C in samples of the two different root size classes were on average 73 and 42% for coarse and fine root samples, respectively (*p* < 0.001). While the proportions decreased with soil depth in coarse root samples, they increased with soil depth in fine root samples (**Figure 4**). Thus, the difference between size classes was largest in the topsoil, where the proportion was almost 2.5-times higher for coarse than for fine roots, but insignificant below 0.5 m depth. This finding was supported by the significant interaction of depth and root size class (*p* = 0.005).

**FIGURE 5:**
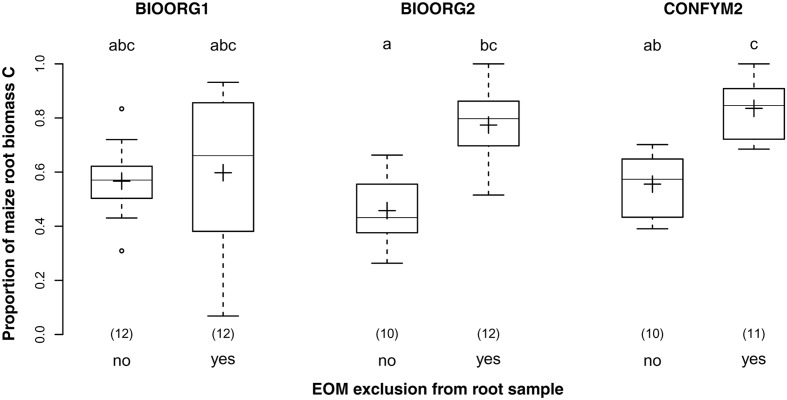
**Proportions of maize root biomass C in root samples from organically (BIOORG) and conventionally (CONFYM) managed plots of the DOK trial with (yes) and without (no) EOM exclusion averaged over soil depth [Combined sample subset: within maize rows, pooled root size class (>0.5 mm)].** Solid line, plus sign, boxes, and whiskers: median, mean, IQR, and 1.5 × IQR, respectively. Differing letters: significant differences between least squares means of proportion values across management systems and EOM exclusion practices at *p* < 0.05. Numbers in brackets: sample sizes.

Exclusion of EOM from the samples increased the proportion of maize root biomass C from 53% in samples that were not cleansed from EOM to 74% in samples for which an attempt of EOM exclusion had been made. Thus, EOM exclusion increased the purity of our samples significantly (*p* < 0.001) but was successful for only about half the amount of EOM. Furthermore, while EOM exclusion did not affect BIOORG1 samples, it increased the purity of BIOORG2 and CONFYM2 samples by 30% each (**Figure 5**) as revealed by the significant interaction of management treatment and EOM exclusion (*p* = 0.003).

## Discussion

### Root Sample Contamination by EOM

Only 60% of the root mass that we retrieved from field soil cores was actual maize root biomass from the current season, the remainder being EOM. The amount of EOM in soil is driven by amount and time of organic inputs, elapsed time until sampling, and conditions for decomposition (Watt et al., 2008). Organic inputs that were likely to contribute to large parts to EOM in our study were roots and stubbles of the preceding grass-clover ley, weed roots, and remnants of manure. Compared to average values over the full crop rotation, inputs of crop roots and manure were very high immediately before maize. With 2.4–4.1 t ha^-1^ of grass-clover ley roots and 2.7–6.4 t ha^-1^ of applied manure, these inputs were almost twice and three times as high as the average yearly inputs of roots (1.6–2.0 t ha^-1^) and manure (1.0–2.4 t ha^-1^), respectively. Furthermore, the elapsed time between these inputs and maize sampling was only 5 months. This indicates that the EOM percentage of 40% found in October 2013 could have been rather high for the DOK site.

The amounts of organic inputs in our study were comparable with organic inputs to maize fields in Swiss agricultural practice. For example, the application rate of farmyard manure to silage maize fields averaged over management systems is approximately 3.3 t ha^-1^ (dry matter) in Switzerland [calculated from Flisch et al. (2009) and Bosshard and Richner (2013)]. Application of organic soil amendments is a common practice in Europe, where about 55% of cropland receive manure as one form of fertilizer, as opposed to the United States, where this percentage is much lower (10%; van Grinsven et al., 2015). The cropping practice in our study is characteristic for Swiss agriculture, indicating that our findings may not be globally applicable.

Numerous studies have reported root sample contamination by EOM (e.g., Ward et al., 1978; Dowdy et al., 1998; Benjamin and Nielsen, 2004; Pietola and Smucker, 2006; Watt et al., 2008) but, to our knowledge, only one study has quantified the amount of EOM in samples also without prior sample cleansing: Watt et al. (2008) measured root length densities of cereals in deep (below ∼0.5 m) soil samples from an agricultural field in Australia which had previously been cropped with canola and wheat and received mineral fertilizer only. By means of cryo-scanning electron microscopy, they identified 21% of total roots as intact cereal (monocot) roots from the current season and the remainder as mainly degraded roots of previous crops or weed (dicot) roots. This finding hints at the possibility that EOM in root samples may play a crucial role also in studies with very different cropping practices than ours.

### Agricultural Management

The proportions of maize root biomass C were statistically similar among management treatments although the average proportions increased slightly with increasing management intensity. As the proportion values express the ratio of recent maize root biomass to total sample mass, similar proportions reflect either similar maize root biomasses in connection with similar masses of EOM or differing maize root biomasses in connection with inversely proportional masses of EOM.

In our study, recent maize root biomasses did not differ significantly between treatments (data not shown) and quantities of EOM must, therefore, also have been similar for all treatments at the time of sampling. However, the amounts of organic inputs differed between treatments: Both root and stubble masses of the preceding grass-clover ley increased with increasing management intensity in October 2012 (data not shown). Similarly, manure application was only about half as high in BIOORG1 as in BIOORG2 and CONFYM2 at the beginning of the maize season. The management effect on the amounts of remnants of the preceding crop and manure at the time they entered soil did therefore not relate to the management-specific proportions of EOM in our root samples. We expect that either another source of organic inputs was contrarily affected by management or decomposition was faster in high than in low intensity treatments or both.

Weed infestation in the DOK trial in 2013 was considerably higher in the bio-organic than in the conventional maize plots (Supplementary Figure S1), which is in accordance with previous findings (Mäder et al., 2002; Rotchés-Ribalta et al., 2016). Furthermore, when nutrients are scarce, weeds are more competitive below ground than domesticated plants that are adapted to high-resource systems (Blackshaw et al., 2004; Kiær et al., 2013). In response to the management treatment and fertilization intensity, we therefore assume that weed root biomass was almost zero in CONFYM2 samples, and lower in BIOORG2 than in BIOORG1 samples. Consequently, the management-specific impact of weed roots on EOM was presumably opposite to that of remnants of the preceding crop and manure. Therefore, the composition of EOM was likely to differ between treatments.

Management-specific decomposition rates have not yet been determined in the DOK trial but might have played a role for the fate of organic inputs in soil. We expect that several management-related drivers interfered with each other and resulted in the insignificant net effect of agricultural management in our study. However, systems with a wide range of organic inputs and turnover dynamics (e.g., sole mineral fertilization, intense weed control, and regular plowing versus sole organic fertilization, full return of straw and green manures, and no tillage) might differ in their amounts of EOM in soil.

### Sampling Depth and Position

The proportion of recent maize root biomass C in root samples from the topsoil was by 8 and 16% lower than the proportions in the two underlying soil depths. We accredit this effect to the predominant occurrence of some EOM components in the topsoil. While plant roots and soil fauna usually appear throughout the profile, organic inputs that are incorporated into soil such as manure and above ground plant residues are confined to the plow layer (Schneider et al., 2006). In this respect, we would have expected the difference to be larger between 0–0.25 and 0.25–0.5 m and less pronounced between 0.25–0.5 and 0.5–0.75 m soil depth. Decomposition dynamics and soil fauna might have led to a more gradual decrease of EOM with soil depth.

Manure and above ground plant residues are preferentially decomposed over roots (Rasse et al., 2005; Zhang et al., 2015) while root decomposition is accelerated by regular physical disturbance and nutrient inputs in the plow layer (Rasse and Smucker, 1998; Turkington et al., 2000). This suggests that organic inputs in the topsoil were not only higher but also disappeared faster than in the subsoil. Furthermore, soil fauna translocate above ground plant residues downward the soil profile. In a controlled experiment where plant seeds were placed near the soil surface, anecic earthworms transported intact seeds to the bottom of the 0.4 m long soil column (Zaller and Saxler, 2007). In the DOK trial after grass-clover ley, anecic earthworms were found to be as abundant as in neighboring grassland strips (Jossi et al., 2007) and might have been relevant for translocation of above ground plant residues.

Topsoil samples taken within maize rows had twice as much maize root biomass C as those taken between rows while subsoil samples did not differ in their proportions of root biomass C. Since sampling position was tested on samples that had been cleansed from EOM, a large part of remaining EOM components presumably comprised hardly detectable roots of the preceding ley and weed. While maize roots usually concentrate in the row, ley and weed roots are more homogeneously distributed in the field (van Noordwijk et al., 1985; Frasier et al., 2016) thus having a greater impact on total root mass between than within maize rows. This effect was primarily visible in the topsoil, suggesting that horizontal distributions of maize roots and ley and weed roots converged below 0.25 m soil depth.

### Root Size Class

The proportions of maize root biomass C in coarse root samples were almost 2.5-times and twice as high as those in fine root samples in 0–0.25 and 0.25–0.5 m soil depth, respectively. As these samples had not been cleansed from EOM, they not only contained intact ley and weed roots but also manure particles, fragments of above ground plant residues, and decaying roots. The purity of coarse root samples was about 74% throughout the soil profile, indicating that mainly non-maize roots contaminated those samples. In contrast, the purity of fine root samples improved from 32% in the topsoil to 58% below 0.5 m soil depth, indicating that those samples were additionally affected by manure and above ground plant residues. This finding was in accordance with Koteen and Baldocchi (2013) who reported that a 1 mm mesh let most of the non-root organic debris pass through.

The sieve size used in our study of 0.5 mm was comparably coarse and did not retain total maize root biomass. Finest roots and root hairs of maize can be as small in diameter as 25 μm (Pallant et al., 1993). While this very fine fraction can amount to the larger part of total root length, its contribution to total root weight is considerably smaller (Oliveira et al., 2000). Livesley et al. (1999) found that a 0.5 mm mesh size retained 94% of the maize root biomass from field soil samples recovered with a 0.25 mm mesh size and considerably reduced the time and effort to separate recent crop roots from EOM. As a consequence, the authors suggested to use two mesh sizes for root biomass determination to circumvent the laborious procedure of cleansing the complete root sample.

### Manual Exclusion of EOM from Root Samples

Exclusion of EOM increased the proportion of recent root biomass C by 30% in row samples of BIOORG2 and CONFYM2, thus reducing EOM to about 40% of its initial amount in these samples. However, this effect was not visible for row samples of BIOORG1 and we again attribute this finding to different EOM compositions in different management treatments. Relative to easily detectable EOM components, BIOORG1 had presumably large amounts of weed roots that could not be distinguished from maize roots by eye. This assumption is supported by the result that two out of 12 BIOORG1 samples had δ^13^C values typical of C3 plants even though EOM had been excluded, indicating that these samples did actually not contain any maize roots. The success rate of root sample cleansing in our study was therefore highly dependent on EOM composition and, at best, about 60%.

However, as Humax and Pürckhauer samples were retrieved and processed differently, this value should be interpreted cautiously. In particular, the kinetic energy of water was higher for automated than for manual root washing and brittle EOM particles may have been broken down and washed out more easily in the root washer than under running tap water. Humax and Pürckhauer samples may have therefore contained different amounts of EOM after washing.

Since root samples taken between rows had less maize root biomass C than those taken within rows, the average proportion of maize root biomass C in all samples, for which an attempt of EOM exclusion had been made, was only 62%. Similarly, Watt et al. (2008) and Pietola and Smucker (2006) reported that only 73 and 80–85%, respectively, of total root length in their cleansed samples consisted of recent crop roots. Although sites, crops, and sample processing procedures differed greatly between the three studies, thorough visual identification and manual removal of EOM by the operators was not sufficient to exclude EOM completely from root samples.

Classical measures to increase EOM detectability in root samples comprise vital staining of the roots and automated image analysis (Richner et al., 2000). While vital staining with, e.g., Congo red or trypan blue is used to differentiate between living and dead roots (Ottman and Timm, 1984), automated analysis of root sample images with predefined shape indices such as a fixed length-to-width ratio mainly discriminates between roots and non-root EOM (Benjamin and Nielsen, 2004; Pietola and Smucker, 2006). Watt et al. (2008) used cryo-scanning electron microscopy of transverse root sections to assign single roots to recent or remnant crops or to weed based on the appearance of cortical and endodermal cells and secondary xylem development. A differentiation approach that does not rely on visual detection is the use of C isotopes, either by taking advantage of natural ^13^C differences between recent crop root biomass and EOM (this study) or by artificially enriching the roots under investigation with ^13^C (Subedi et al., 2006) or ^14^C. As all these methods have their advantages and limitations, the choice of method applied in a study should be according to the specific site condition and research question.

### Additional Methodological Errors in Root Biomass Determination

Other obstacles in root methods can also cause root biomass to be considerably misestimated. As a comparably low cost, low time-intensive method, auger sampling has found wide acceptance in field studies (Oliveira et al., 2000). However, as it only covers a very small area of the field and can take the heterogeneity of horizontal and vertical root distribution only to limited extent into account, it demands many replicates to account for the inherently large variability in data. When sampling is limited to the upper 0.3 m of soil, as much as one third of cereal crop root biomass may be missed as compared to 1 m sampling depth (Fan et al., 2016). Furthermore, when cores are taken at different positions in and between crop rows and core-related root biomasses (in g kg^-1^ soil) are simply averaged between positions (Bolinder et al., 1997), area-related root biomass (in t ha^-1^) can be overestimated by up to 50% (van Noordwijk et al., 1985; Frasier et al., 2016).

Most errors in sample processing in the lab result in an underestimation of root biomass. Storage of soil core samples at room temperature for 1 day, drying at room temperature, and soaking in pyrophosphate before root washing was found to reduce root biomasses of wheat, ryegrass, and sugar beet by up to 40% (van Noordwijk and Floris, 1979; Grzebisz et al., 1989), while freezing before root washing reduced grass root biomass by about one fourth (Price and Heitschmidt, 1989). Root contamination by adhering mineral particles even after thorough root washing can result in root biomass overestimation of up to 60% (Janzen et al., 2002; Richter-Heitmann et al., 2016). Additionally, the choice of sieve mesh size defines root recovery substantially: Two millimeter mesh sieves for root washing were found to retain only about two-thirds and one-third of maize root biomass from top- and subsoil samples, respectively (Livesley et al., 1999), and only about half of wheat and faba bean root biomasses (Amato and Pardo, 1994) as compared to 0.25 and 0.2 mm mesh sieves, respectively.

Methodological specifications of sampling and sample processing can result in severe misestimation of root biomass. The average error of 40% by EOM in our root samples is similar to or even higher than other potential biases. However, as individual errors may overlap, the direction and size of the additive error is hard to predict.

### Implications

Quantification of crop root biomass is necessary for studies of plant response to environmental change, soil C modeling or estimations of soil C sequestration (Bolinder et al., 2007; Fageria, 2013; Paustian et al., 2016). However, one obstacle is clearly the presence of EOM that impedes correct root biomass quantification. As a result, not only recovered root biomass is systematically overestimated, but other root-related traits that are typically linked to root biomass such as root-to-shoot ratios and C rhizodeposition are also at risk of being miscalculated. For example, C rhizodeposition can be estimated by applying fixed root-to-rhizodeposition ratios (Pausch et al., 2013) or by tracking labeled C (^13^C or ^14^C) through the plant into soil and calculating the amount of excess label in the soil relative to the amount of excess label in the roots (Janzen and Bruinsma, 1989). The presence of EOM in root samples does not only result in an inflated sample weight but also dilutes the label enrichment of the sample, resulting in an underestimation of excess label in the roots and, thereby, an overestimation of calculated C rhizodeposition. Furthermore, soil C models may be supplied with incorrect numbers when using root biomass and partly also root exudates as input variables (Parton et al., 1992; Coleman and Jenkinson, 1996). As a consequence, predicted soil C stocks or simulated decomposition rates may be affected.

As EOM in root samples is governed by site (Watt et al., 2008), agricultural management, sampling depth and position, and root size class, it has large implications for all studies that focus on one or more of those factors. For example, the depth-dependency of EOM affects estimations of long-term C sequestration of agricultural sites. As current soil C stocks are related to the amounts of recent gains and losses, the aim to store more C in soils in the future must necessarily imply an increase of inputs or a reduction of outputs or both. Enhanced root systems of annual crops, i.e., more root biomass and deeper roots, is one of the most promising options to increase inputs (Kell, 2012; Paustian et al., 2016) and store C in deeper soil (Kell, 2011; Maeght et al., 2013; Lynch and Wojciechowski, 2015). Existing data of root biomass and distribution would be the basis for assessing the capacity of annual crops to enhance their root systems and soils to store additional C in surface and subsoil horizons. Consequently, the overestimation of root biomass by EOM in general and its depth-dependency in particular would have serious consequences for such calculations.

Although the issue of EOM in root samples has already been addressed decades ago (Schuurman and Goedewaagen, 1971) a universal solution to it is still lacking. Since current analytical approaches are extremely time- and resource consuming (Watt et al., 2008; Koteen and Baldocchi, 2013) and spectroscopic techniques are still highly site-specific (Picon-Cochard et al., 2009; Butnor et al., 2012), future work should focus on the refinement of in-situ measurements of living root biomass by combining different methods that employ spectroscopic and electrical resistance-capacitance measurements (Kell, 2012; Maeght et al., 2013).

## Conclusion

Using the isotopic difference between maize root biomass and non-maize EOM in root samples from an agricultural soil, we identified only 60% of root sample mass as actual recent maize root biomass and the remainder as a mixture of different organic inputs, presumably remnants of the preceding crop, manure, and weed roots. We found a strong effect of both sampling depth and position on the proportion of maize root biomass, whereas the overall effect of agricultural management was insignificant. However, detectability of individual EOM components in the course of manual exclusion of EOM from root samples was strongly affected by management and the success rates were zero for a low intensity system and 60% for two higher intensity systems. We strongly assume that EOM composition governs the effect size of the investigated factors.

Handling total root sample mass as recent crop root biomass may result in severe overestimation of this variable, even more so when root mass data from soil cores of different sampling positions are spatially extrapolated to field scale. Manual exclusion of EOM from root samples may not be adequate to cleanse root samples sufficiently and a generally applicable approach to distinguish between recent crop roots and EOM is urgently needed. The presence of EOM in root samples is still one of the main challenges in root studies on agricultural sites and has severe consequences for soil C modeling and estimations of long-term C sequestration in soil. *In situ* measurement of living root biomass can be a future option to overcome this challenge.

## Author Contributions

JH, SA, and JM formed the concept of the paper. JH, JL, H-RO, and AH collected the data. JH analyzed the data and wrote the manuscript and all authors contributed to the writing of the manuscript.

## Conflict of Interest Statement

The authors declare that the research was conducted in the absence of any commercial or financial relationships that could be construed as a potential conflict of interest.
