# Supplementary material for: Overestimation of Crop Root Biomass in Field Experiments Due to Extraneous Organic Matter
Source: Front Plant Sci. 2017 Mar 1;8:284. doi: 10.3389/fpls.2017.00284 (PMC5331040; doi:10.3389/fpls.2017.00284)
Supplement: Supplementary file 1 [file Data_Sheet_1.DOCX]

Supplementary Material

Overestimation of crop root biomass in field experiments due to extraneous organic matter

Juliane Hirte*, Jens Leifeld, Samuel Abiven, Hans-Rudolf Oberholzer, Andreas Hammelehle, Jochen Mayer

*** Correspondence:** Juliane Hirte: juliane.hirte@agroscope.admin.ch

# Supplementary Data

## Determination of mass ratios of coarse- and fine-to-pooled roots

On each experimental field plot, we excavated in two depths (0 - 0.25, 0.25 - 0.5 m) a half-cylindrical monolith around the bisected rootstock of a maize plant that was grown in a microplot (steel cylinder with 0.55 m length and 0.175 m radius inserted into soil to 0.5 m depth). We took two soil cores with a Riverside auger (Ø 70 mm, Eijkelkamp, Netherlands) below the microplot (0.5 - 0.75 m). Coarse and fine roots were obtained and processed by the same method as those of the Pürckhauer samples but additionally weighed before grinding.

Mass ratios of coarse- and fine-to-pooled (i.e. sum of coarse and fine) roots were then calculated for each pair of coarse and fine roots (mass data not shown). Average mass ratios of coarse-to-pooled roots for the three soil depths (difference between treatments not significant) were 0.42 ± (SD) 0.14 (n = 12) for 0 – 0.25 m, 0.28 ± (SD) 0.06 (n = 12) for 0.25 – 0.5 m, and 0.33 ± (SD) 0.12 (n = 12) for 0.5 – 0.75 m.

# Supplementary Table

Supplementary Table 1: Organic inputs in the topsoil (0 - 0.25m) of organically (BIOORG1 and BIOORG2) and conventionally (CONFYM2) managed plots of the DOK trial that can accrue as EOM in soil. Specific examples and masses (100% dry matter) are given for the crop rotation 2006 - 2012.

| Organic input | Year | Example | Mass [t ha^-1^] |
| --- | --- | --- | --- |
| aboveground residues (straw, stubbles, haulm) of crops and green manure | 2011/12  2010  2009  2008  2007  2006 | grass-clover ley  winter wheat  potato  soybean  winter wheat + green manure  silage maize + green manure | 0.6 – 1.0^a^  0.9 – 1.1^b^  NA^c^  4.2 – 5.3  0.9 – 1.1^b^ + NA  0.6 – 0.9^d^ + NA |
|  | average |  | 1.5 – 1.9 + NA |
| roots of previous crops and green manure | 2011/12  2010  2009  2008  2007  2006 | grass-clover ley  winter wheat  potato  soybean  winter wheat + green manure  silage maize + green manure | 2.4 – 4.1^a^  1.1 – 1.3^b^  NA  1.5 – 1.8^e^  1.1 – 1.3^b^ + NA  1.4 – 1.9^d^ + NA |
|  | average |  | 1.6 – 2.0 + NA |
| organic soil amendments | yearly yearly | manure slurry | 1.0 – 2.4^f^ 0.3 – 1.0^f^ |
| weed | yearly | 30+ different species^g^ | NA |
| soil fauna | yearly | earthworms | 0.3^h^ |

^a^ BIOORG1, BIOORG2, and mineral conventional treatment CONMIN2 (marginally lower intensity than CONFYM2; see Mayer et al., 2015); sampling October 2012, final cut and plowing May 2013
^b^ wheat 2015
^c^ not available
^d^ maize 2013
^e^ 0 - 0.2m soil depth
^f^ average for 2006 - 2012 in t ha^-1^ year^-1^
^g^ Rotchés-Ribalta et al., 2016
^h^ average for 2001 - 2005 in t ha^-1^ sampling^-1^ (Jossi et al., 2007), dry mass estimated from preserved mass at 17 % dry matter content (Lakhani and Satchell, 1970)

Jossi, W., Zihlmann, U., and Dubois, D. (2007). DOK-Versuch: Anbausystem-Effekte auf die Regenwürmer. *Agrarforschung* 14, 66–71.

Lakhani, K., and Satchell, J. (1970). Production by Lumbricus terrestris (L.). *J. Anim. Ecol.*, 473–492.

Mayer, J., Gunst, L., Mäder, P., Samson, M.-F., Carcea, M., Narducci, V., et al. (2015). Productivity, quality and sustainability of winter wheat under long-term conventional and organic management in Switzerland. *Eur. J. Agron.* 65, 27–39. doi:10.1016/j.eja.2015.01.002.

Rotchés-Ribalta, R., Armengot, L., Mäder, P., Mayer, J., and Sans, F. X. (2016). Long-term Management Affects the Community Composition of Arable Soil Seedbanks. *Weed Sci.* doi:10.1614/WS-D-16-00072.1.

# Supplementary Figure


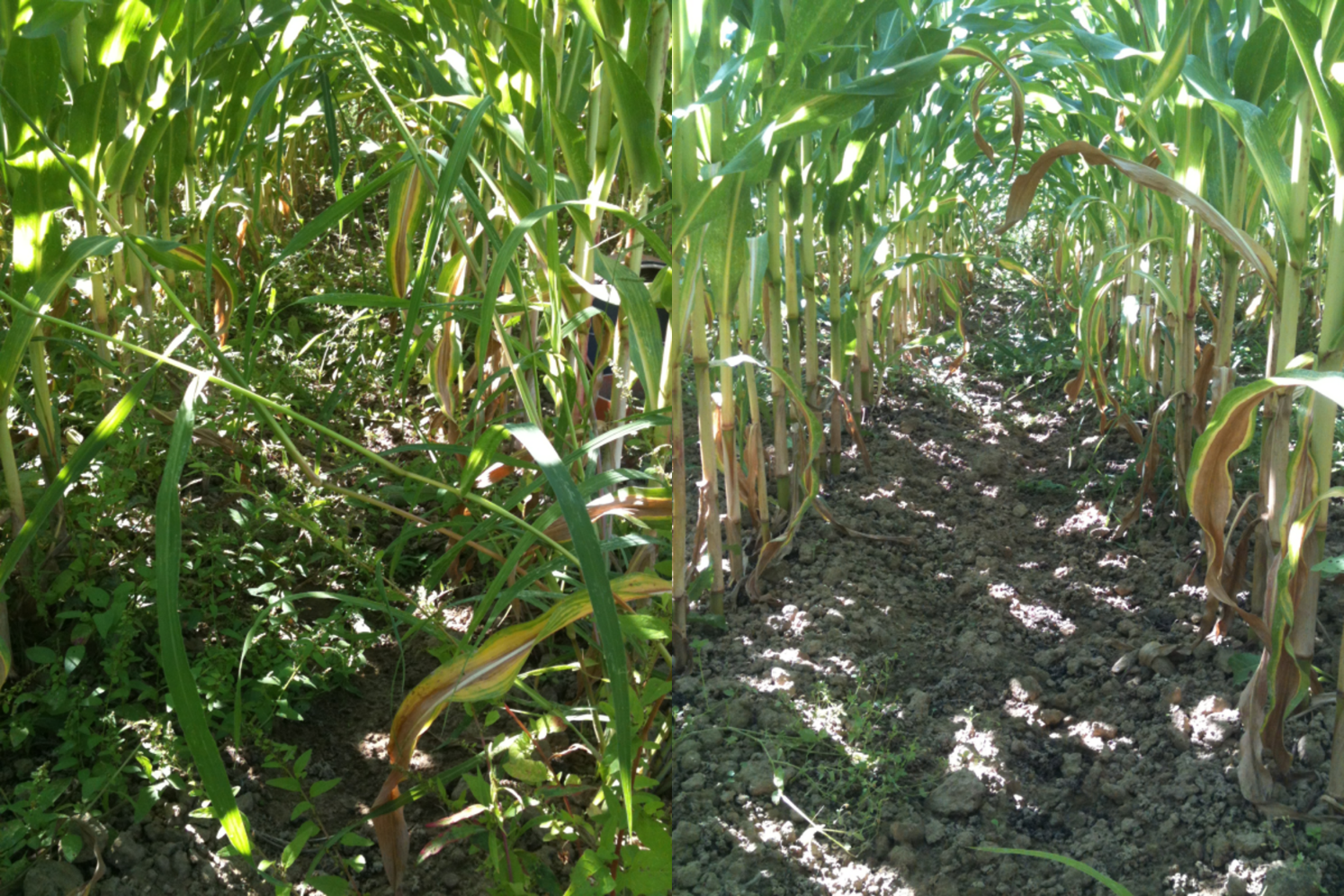


Supplementary Figure 1: Weed infestation in organically (BIOORG2; left) and conventionally (CONFYM2; right) managed maize plots of the DOK trial in September 2013.
